# Impacts and Recovery from Severe Tropical Cyclone Yasi on the Great Barrier Reef

**DOI:** 10.1371/journal.pone.0121272

**Published:** 2015-04-15

**Authors:** Roger Beeden, Jeffrey Maynard, Marjetta Puotinen, Paul Marshall, Jen Dryden, Jeremy Goldberg, Gareth Williams

**Affiliations:** 1 Great Barrier Reef Marine Park Authority, Townsville, QLD, 4810, Australia; 2 Colleges of Business, Law and Governance and Marine and Environmental Sciences, James Cook University, Townsville, QLD, 4810, Australia; 3 Department of Ecology and Evolutionary Biology, Cornell University, Ithaca, New York, 14853, United States of America; 4 Laboratories d’Excellence <<CORAIL>> USR 3278 CNRS—EPHE, CRIOBE, Papetoai, Moorea, Polynésie Francaise; 5 School of Earth and Environmental Sciences, University of Wollongong, Wollongong, NSW, 2522, Australia; 6 Australian Institute of Marine Science, Perth, WA, 6009, Australia; 7 Reef Ecologic, 10 Mt. Clifton Court, Alligator Creek, QLD, 4814, Australia; 8 CSIRO Land and Water Flagship, Townsville, QLD, 4811, Australia; 9 Center for Marine Biodiversity and Conservation, Scripps Institution of Oceanography, La Jolla, California, 92037, United States of America; Universidad Nacional Autónoma de México, MEXICO

## Abstract

Full recovery of coral reefs from tropical cyclone (TC) damage can take decades, making cyclones a major driver of habitat condition where they occur regularly. Since 1985, 44 TCs generated gale force winds (≥17 metres/second) within the Great Barrier Reef Marine Park (GBRMP). Of the hurricane strength TCs (≥H1—Saffir Simpson scale; ≥ category 3 Australian scale), TC Yasi (February, 2011) was the largest. In the weeks after TC Yasi crossed the GBRMP, participating researchers, managers and rangers assessed the extent and severity of reef damage via 841 Reef Health and Impact Surveys at 70 reefs. Records were scaled into five damage levels representing increasingly widespread colony-level damage (1, 2, 3) and reef structural damage (4, 5). Average damage severity was significantly affected by direction (north vs south of the cyclone track), reef shelf position (mid-shelf vs outer-shelf) and habitat type. More outer-shelf reefs suffered structural damage than mid-shelf reefs within 150 km of the track. Structural damage spanned a greater latitudinal range for mid-shelf reefs than outer-shelf reefs (400 vs 300 km). Structural damage was patchily distributed at all distances, but more so as distance from the track increased. Damage extended much further from the track than during other recent intense cyclones that had smaller circulation sizes. Just over 15% (3,834 km^2^) of the total reef area of the GBRMP is estimated to have sustained some level of coral damage, with ~4% (949 km^2^) sustaining a degree of structural damage. TC Yasi likely caused the greatest loss of coral cover on the GBR in a 24-hour period since 1985. Severely impacted reefs have started to recover; coral cover increased an average of 4% between 2011 and 2013 at re-surveyed reefs. The *in situ* assessment of impacts described here is the largest in scale ever conducted on the Great Barrier Reef following a reef health disturbance.

## Introduction

Extreme winds during tropical cyclones (TCs—also known as hurricanes, typhoons) generate heavy seas that can devastate coral reef communities [1,2], which can buffer human communities along coasts from the sea conditions that TCs generate. The types of damage to corals and reefs include breakage of coral colony tips and branches, sand burial, dislodgement of large colonies, and structural damage where sections of the reef framework are partly or wholly removed [1,3,4]. Recovery may take decades to centuries [2,5,6] in cases of structural damage assuming access to a sufficient larval pool [7,8]. When such damage reoccurs frequently enough, especially in combination with other disturbances and anthropogenic stress, coral cover may be lowered sufficiently to threaten the ability of reefs to sustain themselves as coral-dominated systems [9–13]. For example, recurrent cyclones combined with overfishing and coral disease have been a major driver of the decline of reefs in the Caribbean over the last three decades.

The Great Barrier Reef (GBR) is regularly exposed to gale force (≥17 metres/second: m/s) or higher winds generated by TCs, averaging 4 days per year from 1985 to 2009 in the central GBR [14]. Where such TCs are intense or long-lasting enough or both, the heavy seas they generate can cause structural damage to coral reefs, as was recorded in field surveys after intense TCs Ivor in 1990 [3] and Ingrid in 2005 [4]. Similar damage was also observed at Jamaican reefs [15] following Hurricane Allen, which was H4 on the Saffir-Simpson scale while near the surveyed reefs. These high-energy events leave a lasting legacy in the geological record, producing storm ridges that can be preserved for thousands of years [1]. Dating of such ridges throughout the GBR and adjacent coast provides evidence of repeated TC structural damage over the past 5,000 years [16]. Cyclones are a major driver of habitat condition in the GBR—De’ath et al. [17] attribute nearly half of the observed coral loss across the GBR from 1985–2012 to wave damage caused by TC-generated winds.

TC intensity is only one factor affecting the potential to generate heavy seas capable of damaging coral communities. More intense TCs create faster maximum winds and higher maximum wave heights than less intense cyclones and are characterised by lower central pressures [18,19]. However, the overall area encompassed by a cyclone’s circulation may be more important in determining the total destructiveness of the TC [20]. For a given intensity, large TCs extend extreme conditions over much greater distances than small TCs (100s vs 10s of km [21,22]). In general, intense TCs can be any size [21], though near the northeast Australian coast, TC intensity tends to peak when TCs are small [22]. Several recent studies of TC damage to coral reefs assume that structural damage is generally not found beyond a distance to the track determined by cyclone intensity ([23]–100 km; [24]– 90km) or by cyclone intensity and side of the track ([25]– 160 km). To date, there are no published studies presenting field survey data on the spatial extent of wave damage to coral reefs from a TC that is both large and intense.

**Fig 1:**
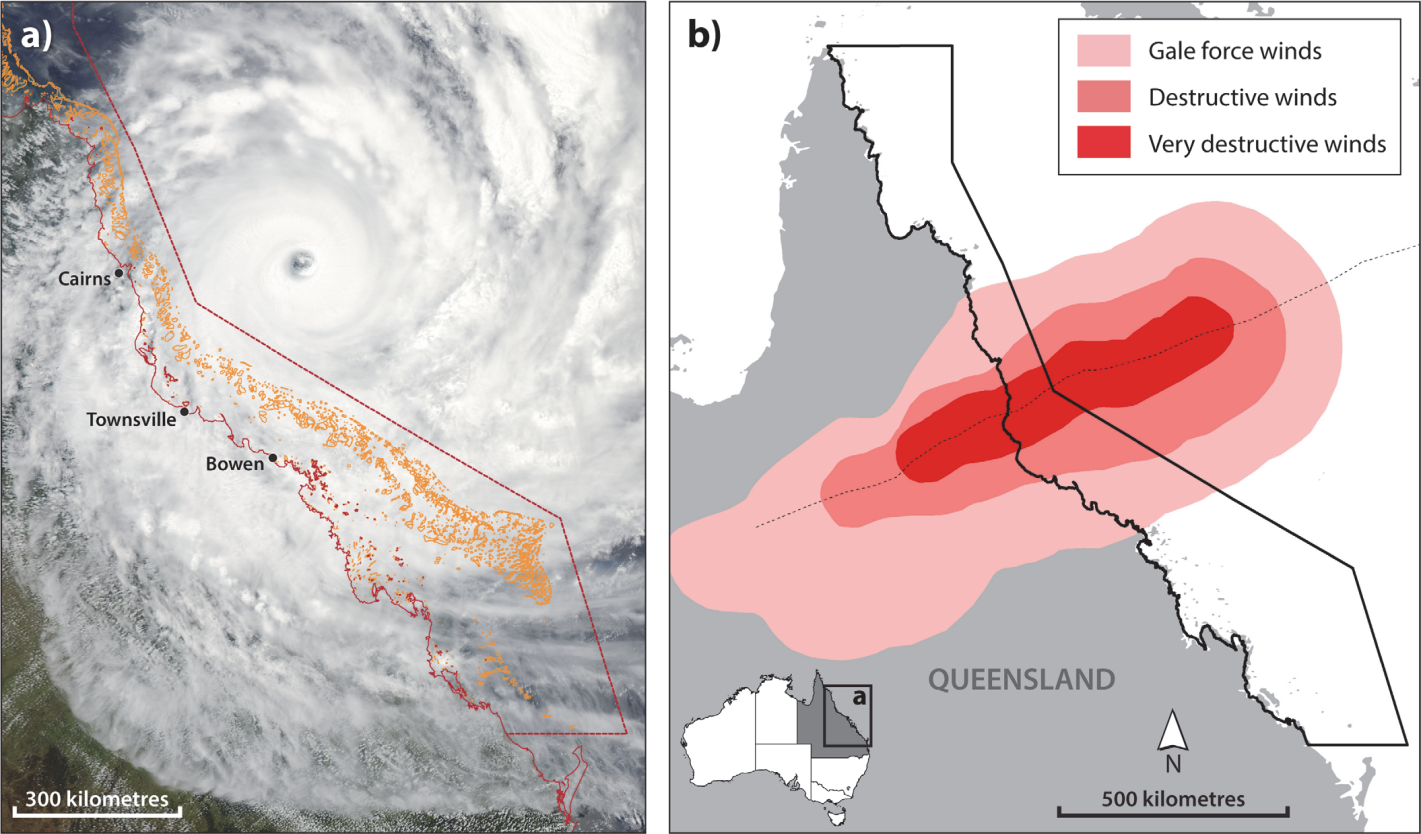
Spatial extent of TC Yasi with wind zone boundaries. (a) Satellite-based photograph of TC Yasi on February 2, 2011 prior to crossing the Queensland coast between Townsville and Cairns on February 3 (images courtesy of the Australian Bureau of Meteorology (BoM)). (b) Boundaries of gale force, destructive and very destructive winds from BoM; the extent of gale force winds north to south along the GBR exceeded 600 km.

Initially forming in the Coral Sea as a tropical low on 29 January 2011, TC Yasi intensified to hurricane force (wind speeds ≥33 m/s) at 700 UTC on January 31. Maximum wind speeds were estimated to be 215 km/hr (59.7 m/s) with gusts up to 285 km/hr (79.2 m/s) and a minimum central pressure of 929 hectopascals when TC Yasi crossed the Queensland coast at 1400 UTC on 3 February. TC Yasi made landfall with the highest intensity of any TC that crossed the Queensland coast since 1918 [26]. With an estimated extent of gale force winds more than 600 km wide, Yasi was also notably large (Fig. 1), posing challenges for assessing the spatial extent of the damage caused to coral reefs. However, when TC Yasi crossed the coast, the Great Barrier Reef Marine Park Authority (GBRMPA) had just finished incorporating mechanical damage into the Reef Health and Impact Survey (RHIS) protocol used in the Eye on the Reef participatory monitoring network [27]. Training managers, rangers and participating researchers to use the protocol meant that a large number of reefs could be rapidly assessed spanning the entire potentially impacted area.

The impact assessment undertaken following TC Yasi is the most spatially extensive field survey of TC impacts on coral reefs ever conducted and reported on (841 surveys of 70 reefs, ~10% of the 775 reefs within the gale force wind boundary, Fig. 1). Other published field surveys after TC Yasi looked for evidence of damage at only 2 [28] or 4 reefs [29]. We first compare the characteristics of TC Yasi with other TCs that have generated gale force winds in the GBR Marine Park (GBRMP) between 1985 and 2014. This analysis sought to answer this question: Was TC Yasi unique among TCs present in the GBRMP between 1985 and 2014, and if so, in what way? We then present and discuss the results of the impact assessment and subsequent recovery surveys, which sought to answer these three research questions:

1. What are the spatial patterns in damage severity throughout the area affected by gale force winds during TC Yasi?
2. Do the spatial patterns in damage vary significantly with direction and distance from the cyclone eye and with shelf position and habitat?
3. What are the spatial patterns in recovery of live coral between 2013 and 2011 and what are the changes during that timeframe in the cover of recently dead coral, live coral rock, coral rubble, sand and macroalgae.

## Materials and Methods

Data from the Australian Bureau of Meteorology were used to compare the key characteristics of TC Yasi to each of the 43 other TCs that produced gale force winds in the GBRMP between January 1985 and December 2014 (n = 44). The potential for a TC to cause structural damage to reefs is driven by three key factors: the intensity, circulation size and the duration of extreme conditions near reefs (‘persistence’). We classified each cyclone based on maximum intensity (maximum surface wind speeds in m/s), average size (mean radius from the eye of the storm to the outer edge of gale force winds in km), and persistence (total hours of gale force or higher winds within the GBRMP). TCs with ‘severe’ intensity are those that reach hurricane force (wind speeds ≥33 m/s; central pressure <970 hPa; H1 on the Saffir Simpson scale, category 3 on the Australian cyclone scale). The mean gale radii of ‘large’ TCs exceeded 300 km while the radii of ‘small’ TCs were less than 150 km [21]. For severe TCs, the average gale radii was measured for only those eye positions when the TC was severe. Gale force wind duration was estimated by finding the cyclone-generated wind speed across the GBR every hour during each TC and counting the number of hours it met or exceeded gale force conditions. For each TC, values for the three variables were scaled to the maximum recorded value over the time period while present in the GBRMP (intensity—70 m/s; size—495 km; persistence—85 hours). The resultant values were multiplied to create an index for the likelihood the storm caused structural damage, which we call ‘structural damage risk’. The following categories are used in describing structural damage risk: very low to none (index values less than 1), low (index values 1–5), moderate (index values 5–10) and high (index values 10 or more). As the index measures persistence within the GBRMP rather than near specific reefs, it is possible for a TC to be very persistent in an area reasonably far from reefs. Manual adjustments to index categories were made in a few cases to adjust for this. On our conceptual diagram, each TC’s intensity (x axis), circulation size (y axis), persistence (boldness of circles) and structural damage risk value (colour of circles) are plotted.

**Fig 2:**
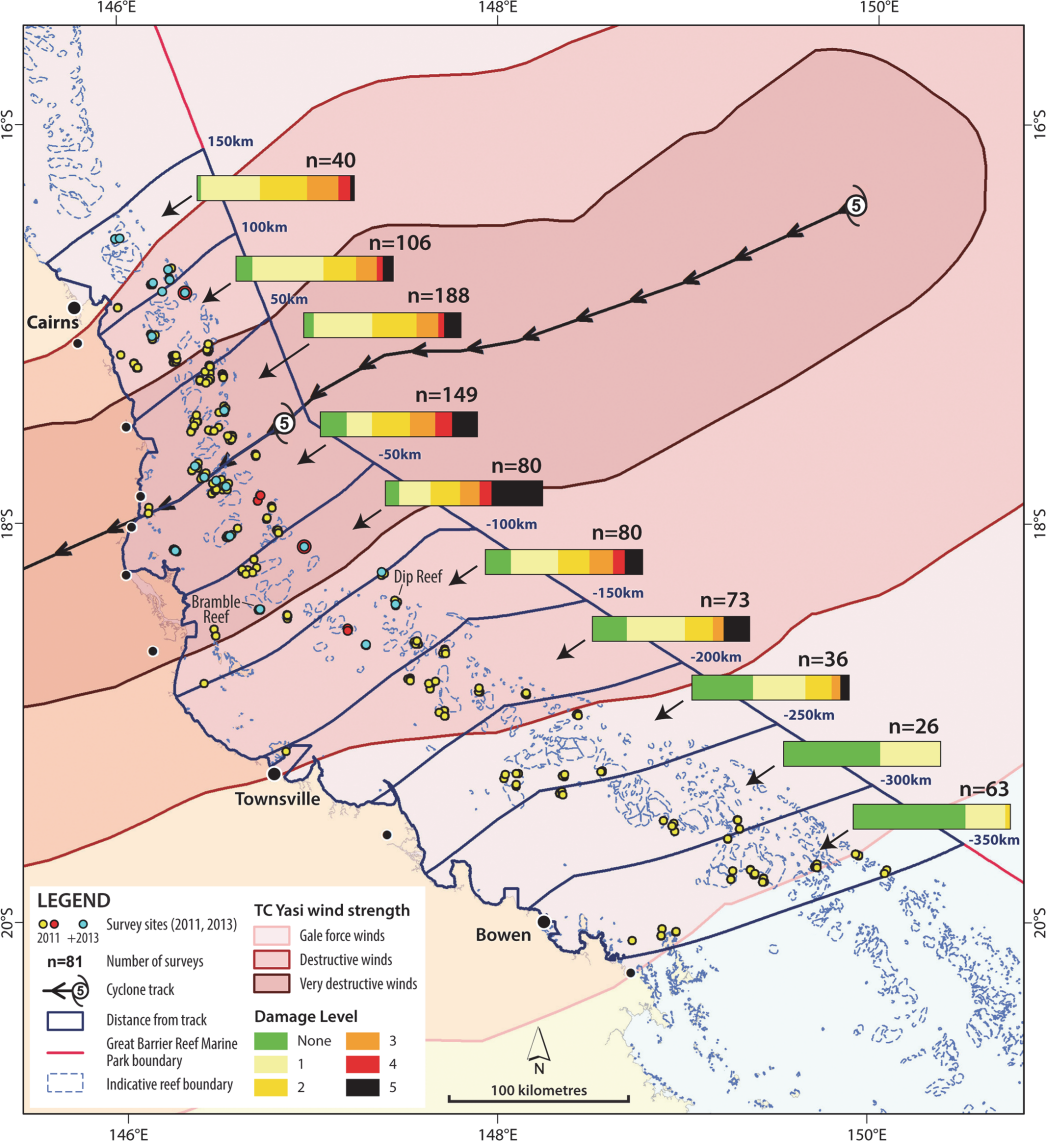
TC Yasi survey reef locations (surveyed February 10 to March 17, 2011 and from January 1 to September 30, 2013). Bar charts for each 50-km Marine Park segment north and south of the track of TC Yasi represent the proportion of surveys that recorded each of 5 levels of damage (n = 841). Locations in red denote reefs where ≥60% of the surveys recorded structural damage (level 4 or 5) and the two labeled reefs are the locations in Fig. 8. Locations in blue were surveyed during both 2011 and 2013. See Figs. 3 and 4 for damage level descriptions.

Reef Health and Impact Surveys (RHIS) were used to document the geographical extent, severity and patchiness of damage to reefs exposed to extreme winds (and consequently rough seas) during TC Yasi (Fig. 2). RHIS is a rapid survey method jointly developed by the GBRMPA and the Queensland Parks and Wildlife Service (QPWS) [27]. To undertake a RHIS survey, observers first select a location, swim to find the habitat type they intend to survey and then do several fin kicks with their eyes closed to randomly select a starting point. The survey area is a circle with a 5 m radius, so a 5 m swim is made to four points from the centre; like the N, S, E and W of a compass. Those points form the circle perimeter and observers swim the circumference while looking into the survey area to estimate the percent cover of the substrate made up by the various benthic groups. Coral and macroalgae are then classified by life form and type, respectively, and then observers focus on signs of impacts and their potential causes [27].

**Fig 3:**
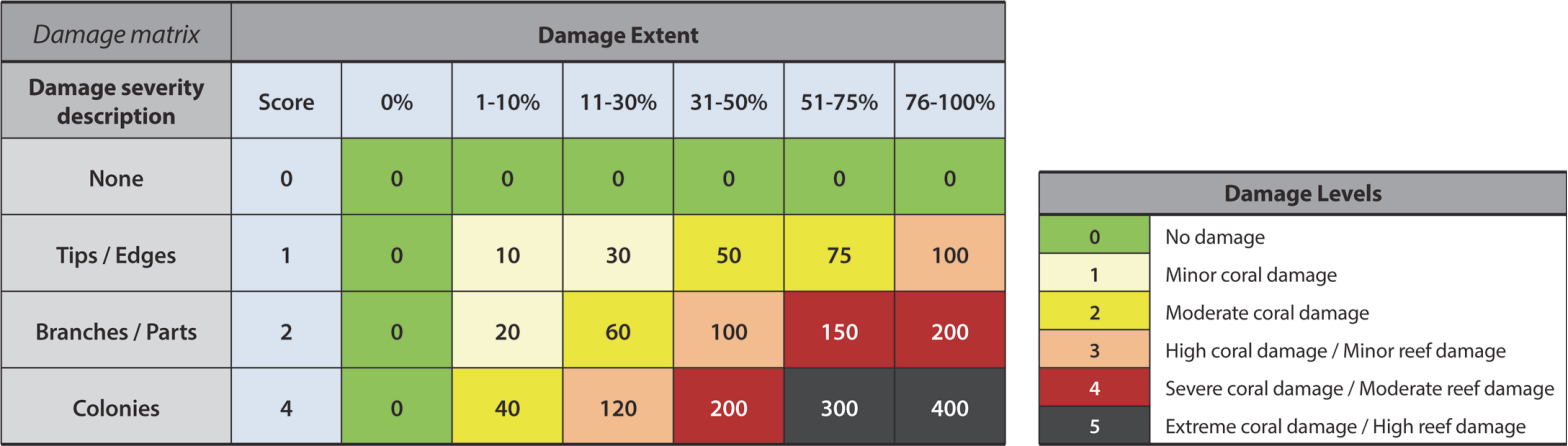
Cyclone damage matrix. Damage extent and severity scores in light blue represent the survey area damaged (Damage extent) and the dominant type of colony-level damage observed (Damage severity description). Damage levels 1, 2 and 3 relate to coral damage, while 4 and 5 relate to reef structural damage (see colour scale on right). Representative photographs of each damage level are shown in Fig. 4 with damage descriptions.

**Fig 4:**
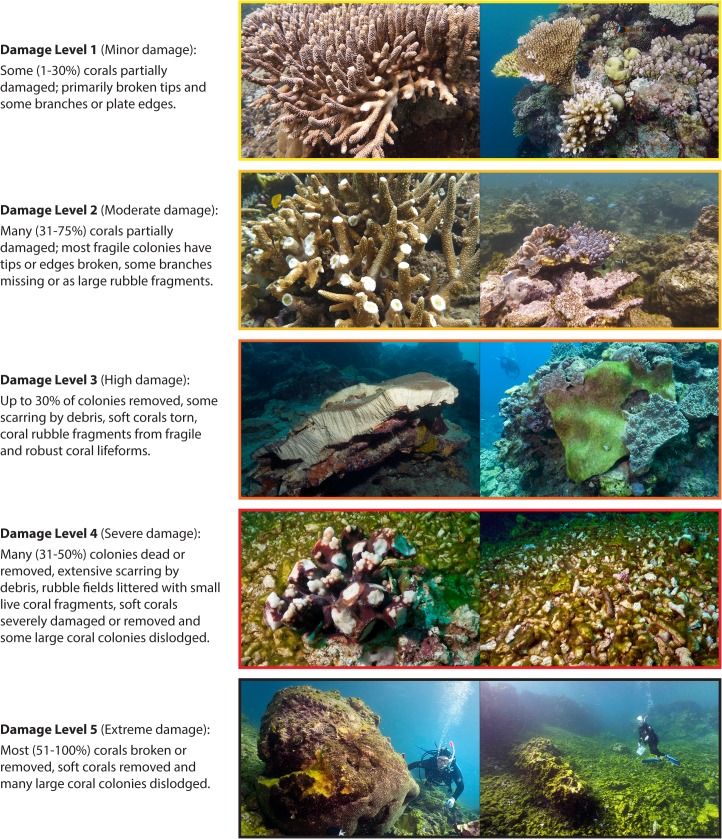
Representative photos of the 5 damage levels used in the impact assessment and analysis. The damage levels used follow the matrix in Fig. 3, which combines damage extent with the dominant type of colony-level damage observed.

For these assessments, team members estimated the proportion of coral cover damaged and classified the most common level of impact severity observed as one of the following: None, Tips/Edges, Branches/Parts, and Colonies. A damage impact matrix was developed to integrate the extent and severity scores for each survey into one of five levels of damage (Fig. 3). The matrix and damage levels were developed to be comparable to those developed by AIMS to assess the impact of TC Ingrid [4]. The five damage levels used encapsulate both colony and reef damage. Damage Levels 1 and 2 indicate partial colony mortality. Damage Levels 3, 4 and 5 indicate the increasing extent of complete colony mortality and reef framework damage. Of these, levels 4 and 5 are referred to throughout as structural damage. Fig. 4 presents photographs of damage that are representative of each of the five damage levels.

Teams from GBRMPA, QPWS, the Australian Institute of Marine Science (AIMS), and the tourism and fishing industries completed 841 surveys at 70 reefs within five weeks (between 10 February and 17 March 2011) of TC Yasi crossing the Queensland coast (3 February 2011, Fig. 2). The reefs surveyed in 2011 included nearly 10 percent (70 / 775) of those located within the gale force, destructive or very destructive wind boundaries (see Fig. 1B) and three reefs beyond these zones (Fig. 2). Surveys spanned the continental shelf (inner—12, middle—34, outer—24 reefs), and extended from 150 km north (weak side) to 350 km south (strong side) of the track of the cyclone eye. Geographic coordinates for the surveyed reefs are available with our data via this DOI (doi:10.5061/dryad.3gn80). Teams completed at least three surveys for at least three locations at each reef, including these habitat types: lagoon (1–3 m in depth), reef flat (1–3 m), crest (~5 m), slope (7–10 m) or bommie fields (7–12 m). All required permits for scientific surveys were obtained from the Permits section of the Great Barrier Reef Marine Park Authority. Surveys were undertaken on snorkel and SCUBA.

Survey data were collated in 50 km segments from 150 km north to 350 km south of the track (10 segments). Within these segments, the percentage of surveys that recorded each of the five damage levels was calculated. To estimate the area of coral reef affected at each damage level, the proportion of each level of cyclone damage observed during the surveys was extrapolated to the known reef area within each of the ten 50 km segments. Estimates were then produced of the percentage of the total reef area within the Marine Park (24,839 km^2^) impacted by TC Yasi at each damage category level. Data were aggregated at the scale of individual reefs to identify the percentage of surveys at each reef for which structural damage was observed.

Damage levels for surveys within each 50 km segment were also averaged and a custom coded bootstrap routine with R 2.15.3 (R Development Core Team, www.R-project.org) was used to generate 95% confidence intervals using resampling with replacement (10,000 times) of the ten 50 km segments. This method generates estimates of error while accounting for unequal effort among the 10 segments and pools all data (no discrimination between habitat or shelf position). A permutational analysis of variance (PermANOVA) was also used to test the effects of three fixed factors on the mean level of damage experienced: side of track (two levels: north/south of the eye), shelf position (two levels: mid-shelf and outer-shelf), and habitat (5 levels: lagoon, reef flat, crest, slope and bommie fields). The independent effects of each factor and their possible interactions and all post-hoc pairwise comparisons across levels were calculated using PERMANOVA+. This analysis is based on a Bray-Curtis similarity matrix, 9999 permutations of the raw data under a reduced model, and Type III (partial) sums-of-squares.

Recovery surveys were conducted between January and December 2013 (24–35 months after TC Yasi) at reefs from 150 km north to 150 km south of the cyclone track (the area where damage was most severe). The recovery surveys were conducted in the same manner as the impact assessments at a sub-set of the reefs surveyed in 2011 (see blue locations in Fig. 2). The number of reefs re-surveyed in 2013 in each of the 50 km segments is as follows (150 km—n = 3, 100–3, 50–3, -50–4, -100–3, -150–2, -200–1). Reefs are only included where at least 3 surveys were completed during each survey year. Percent change was calculated for live coral, recently dead coral, live coral rock, coral rubble, sand and macroalgae. Values for percent changes are aggregated at the scale of individual reefs for comparison between the two years. We then calculated the average values and standard deviation within each of the 50 km segments.

## Results and Discussion

**Fig 5:**
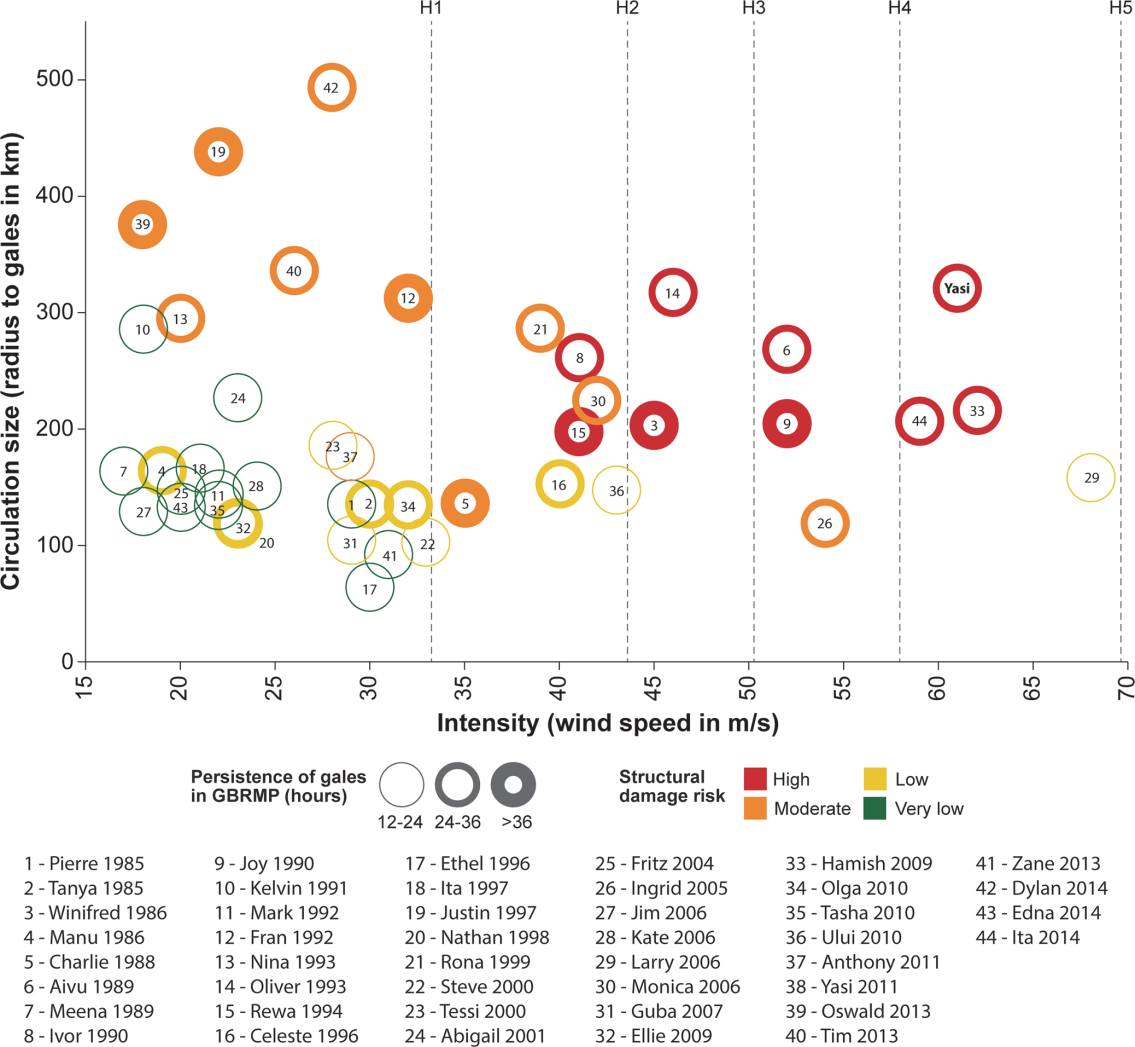
Potential for structural damage (very low to high) of GBR coral communities from tropical cyclones (TCs, 1985–2014). The 44 TCs are plotted on the diagram with respect to their intensity along the horizontal axis and their circulation size along the vertical axis. Of the 7 TCs that were large, only TC Yasi was also intense and posed a high risk of structural damage.

Since 1985, TC Yasi was the only cyclone to generate gale-force winds within the GBRMP when both intense and large, and the only large TC to pose a high structural damage risk (Fig. 5). TC Oliver (1993) affected the far southern GBR when large and intense, but was located far from all but a few reefs. Nearly a third (30%, n = 13) of the 44 TCs that produced gale force winds in the GBR from 1985–2014 posed a very low risk of causing structural damage (Fig. 5). These storms did not generate extreme sea conditions near reefs because they were not sufficiently intense or large or were not sufficiently persistent near reefs. Just over one-third (39%, n = 17) of the TCs in the study period were intense and more than half (64%, n = 28) were persistent within the GBRMP (Fig. 5). TCs much more intense than TC Yasi (central pressures ≤ 920 hPa versus Yasi’s minimum hPa of 929) track within the GBR only once every ~200–300 years (~0.5% probability of occurrence in a given year [16,30]).

The extensive field data set we collected following TC Yasi demonstrates that a single large and intense cyclone can cause structural damage over a vast area within a very short timeframe (~ a day). Damage was observed throughout the area of the Marine Park exposed to gale force, destructive and very destructive winds during TC Yasi—an area spanning approximately 4 degrees of latitude (Figs. 1, 2). Damage from TC Yasi ranged from minor tissue injuries at the edges and tips of fragile coral colonies to total removal of all sessile organisms and abrasion and fracturing of the reef substrate. In total, just over 15% (3,834 km^2^) of the 24,839km^2^ reef area within the GBR Marine Park is estimated to have sustained some level of coral damage, with 4% (949 km^2^) of reef area sustaining severe coral damage and some degree of structural damage (Table 1). Structural damage (levels 4 and 5) extended as far as 150 km to the north and 250 km to the south of the cyclone track. This demonstrates that structural damage from an intense and large TC can extend farther than typically reported distance thresholds for structural damage (90 km [24], 100 km [23], 160 km [25]).

**Table 1:** Total and percentage reef area affected within each damage level. Damage levels are described in Figs. 2 and 3.

Damage Level	Damage Level Descriptions	Total Reef Area Affected (km^2^)	Proportion of Affected Reef Area Within the Marine Park (%)
**Level 0**	No Damage	21,005	84.5
**Level 1**	Minor Coral Damage	1,388	5.6
**Level 2**	Moderate Coral Damage	933	3.8
**Level 3**	Severe Coral Damage	564	2.3
**Level 4**	Severe Coral Damage and Moderate Structural Damage	447	1.8
**Level 5**	Extreme Coral Damage and High structural Damage	502	2.0

At locations with structural damage (levels 4 and 5, Figs. 2 and 3) few corals escaped substantial physical injury, and many were so severely damaged that only their bases remained attached to the reef. The majority of large soft corals either suffered substantial tissue loss or were completely removed, as indicated by layers of spicules formed where the coral was attached to the substrate. Extensive fields of freshly formed rubble were seen, including large numbers of coral fragments still covered in live tissue. At the worst affected locations, extensive areas of reef structure were completely scoured. Few corals or other sessile organisms remained attached to the reef structure and some really large corals likely to be hundreds of years old had been dislodged and overturned (picture example Fig. 4).

Structural damage to corals from TC Yasi was spatially extensive. Overall, 24% of the surveys recorded structural damage, and some incidence of structural damage was found at 66.2% of the reefs that were surveyed. The spatial distribution of structural damage was highly patchy, as would be expected [2] even for an intense TC (for example, the same was observed for TCs Ivor [3] and Ingrid [4]). Only four (5.3%) of the surveyed reefs showed structural damage for 60% or more of the surveys. These more uniformly devastated reefs were Pellowe, Unnamed 17–065, Unnamed 18–023 and Hopkinson (see red, Fig. 2). They fell within the highly destructive and destructive wind zones defined by the Australian Bureau of Meteorology (Fig. 1), ranging from 100km north to 150 km south of the track and include 1 mid-shelf reef and 3 outer-shelf reefs. At these reefs, remaining coral cover after TC Yasi was typically less than 20% (Pellowe—33%, UN17-069–17%, UN18-023–19%, Hopkinson -14%).

**Fig 6:**
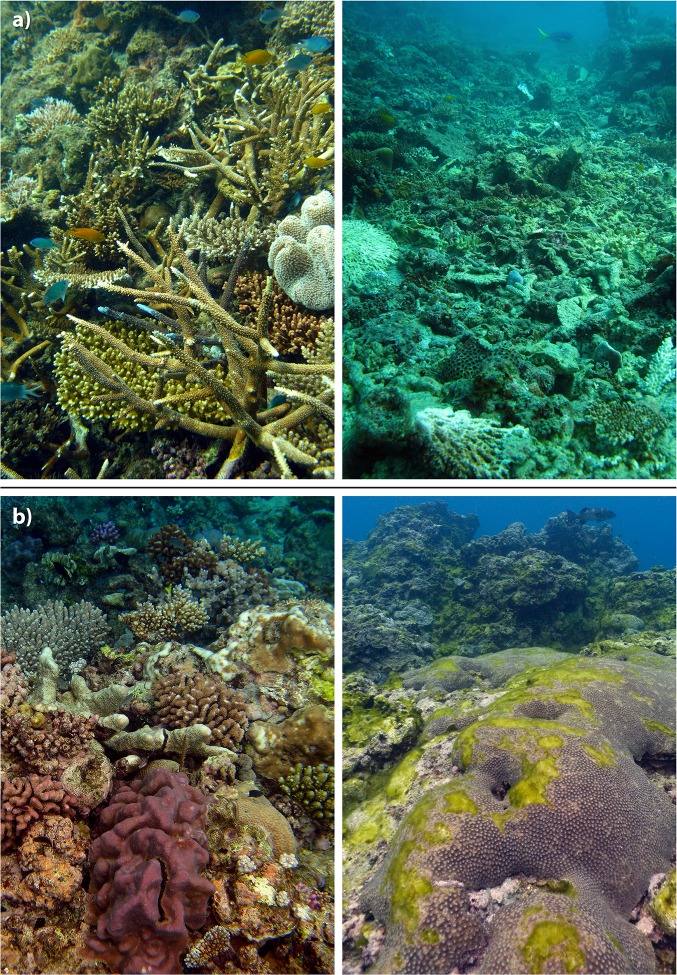
Photos taken within 50 m showing the patchiness of damage at Bramble Reef in the very destructive wind zone and Dip Reef in the destructive wind zone. See Fig. 1 for destructive and very destructive wind zone boundaries and Fig. 2 for reef locations.

In both the very destructive and destructive wind zones (Fig. 1) there were some reefs where structural damage was observed within 50 m of reef that escaped completely unscathed (Fig. 6), which is not suprising. The amount of TC-generated wave energy actually reaching a given part of a reef within the complex GBR setting depends on the location of other nearby reefs and land given the incoming wave direction and the tide [31]. The wave energy is then transformed as it interacts with the variable topography characteristic of reefs and their colonies [32] with some areas far more exposed to extreme conditions than others even if the areas are in close proximity. In addition, the likelihood of structural damage from a given level of wave energy is greater where colonies of mechanically vulnerable shapes are prevalent [33], especially when such vulnerability rises with colony size [34].

**Fig 7:**
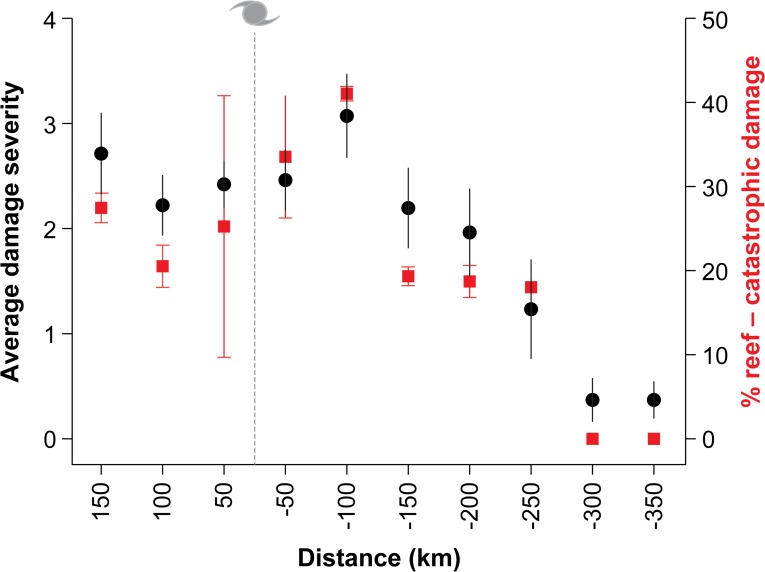
Average damage severity (with 95% confidence intervals) in each of the surveyed 50 km segments of the Marine Park (see Fig. 2, surveys February-March, 2011). The percent of reef with structural damage (damage level of 4 or 5, Figs. 3 and 4) is also shown (±SE). The cyclone symbol denotes the location of the track of the cyclone eye.

Average damage severity did not differ significantly between any of the 50 km segments from 150 km north to 200 km south of the cyclone track (Fig. 7). Average damage peaked 100 km south of the track (3.09±0.19) but was not significantly greater there than for 150 km north (2.73±0.18). The proportion of reefs with structural damage was roughly equal for all three 50 km segments north of the track (24, 21 and 23% for 50, 100 and 150km segments, respectively). Within 50 and 100 km of the cyclone track the prevalence of high coral damage and structural damage was greater to the south (high—33%, structural—47% of surveys) than to the north (high—24%, structural—21%) of the track (Fig. 7). South of the cyclone track, average damage (± 95% confidence interval) rapidly declined as distance from the track exceeded 200 km (Fig. 7). Accordingly, the proportion of surveys recording minor damage or no damage increased with distance south of the track, from 33% of reefs up to 50 km south to >90% beyond 250 km (Fig. 7). No structural damage was recorded for surveys of reefs south of 250 km (Fig. 7).

**Fig 8:**
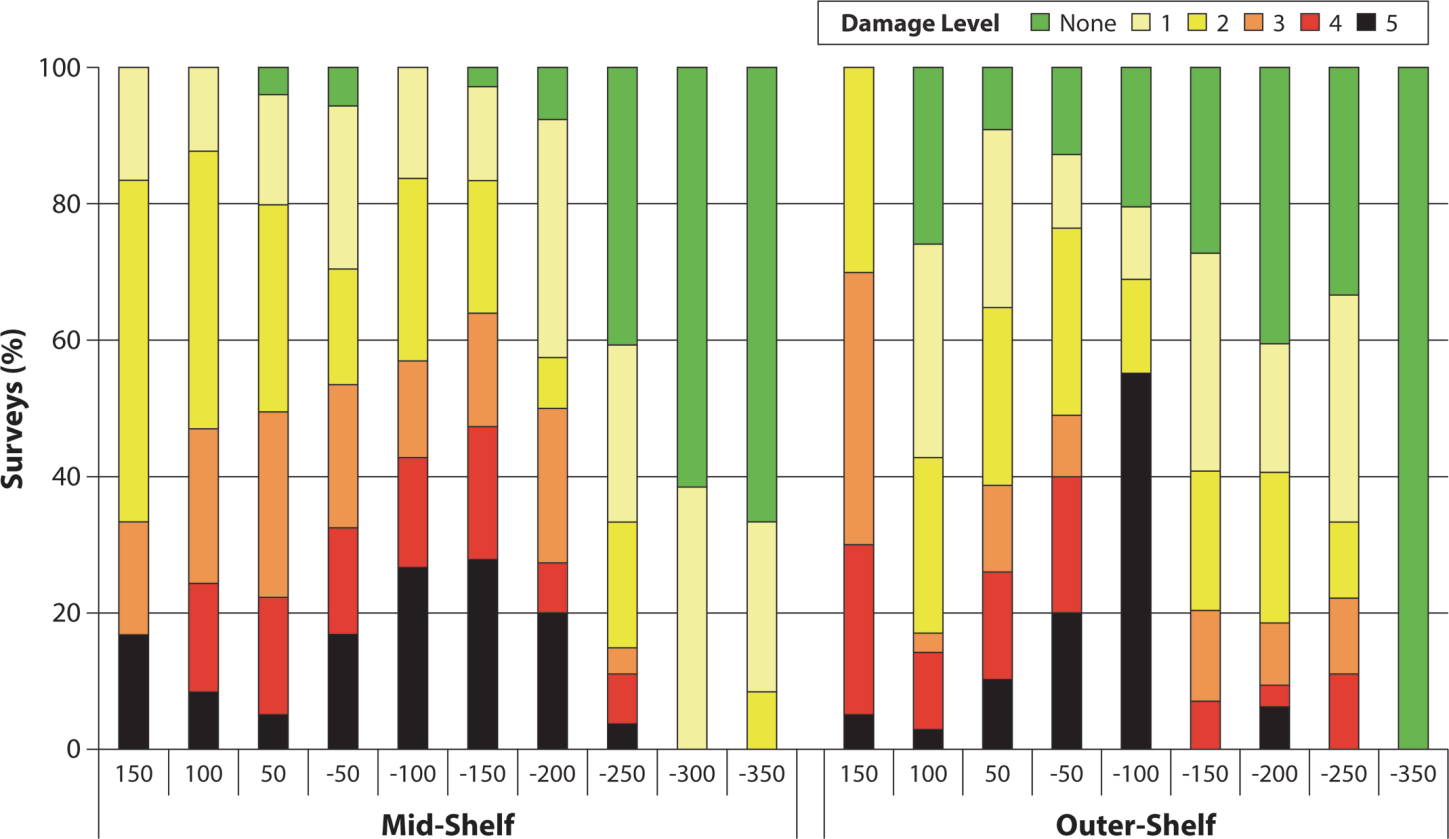
Proportion of surveys that recorded each level of cyclone damage at mid-shelf and outer-shelf reefs within each 50 km segment (Fig. 2) of the Marine Park. Damage level descriptions can be found in Figs. 3 and 4.

Aside from distance from the track (reviewed above), side of track, shelf position, and habitat all significantly influenced the severity of damage observed. There was a significant interaction between the side of track, shelf position, and habitat in the PermANOVA (Pseudo-F_5,754_ = 3.2054, p = 0.003). In pairwise comparisons to determine interactions, side of track and habitat were significant (Pseudo-F_5,754_ = 3.8805, p<0.01) as were habitat and shelf position (Pseudo-F_5,754_ = 2.1667, p<0.05) but not side of track and shelf position. The prevalence of the worst structural damage (level 5) peaked at outer shelf reefs south of the track in the 150 km segment, where more than half of surveys detected it. This was double the prevalence of such damage in the 150 km segment north of the track (Fig. 8). This is in keeping with sea states generated by TC winds being most extreme south of cyclone tracks; a a feature of tropical cyclones (southern hemisphere). At least one instance of severe structural damage was observed at a greater number of mid-shelf (17) than outer-shelf (13) reefs located between 150 km north and south of the track. The total number of surveys that recorded structural damage was higher for mid-shelf (47) than outer-shelf (38) locations. This may be due to mid-shelf reefs serving as a barrier for outer-shelf reefs south of the cyclone track given mid-shelf reefs reduce the fetch for outer-shelf reefs for westerly winds. At outer-shelf reefs north of the cyclone track, lagoon habitats, reef flats and reef crests all suffered more damage than reef slopes, which may have been protected due to their slightly greater depth (10–15 m versus 1–7 m) (3.269, 2.857, and 2.778 vs. 1.643; t = 4.96, p<0.001; t = 3.12, p<0.01; t = 2.34, p<0.05 [respectively]). However, there were no significant differences in average damage between habitats for mid-shelf reefs north of the cyclone track. There were also no significant differences in average damage between habitats for outer-shelf or mid-shelf reefs south of the cyclone track, meaning the slightly greater depth of reef slopes only reduced damage severity at some locations.

**Fig 9:**
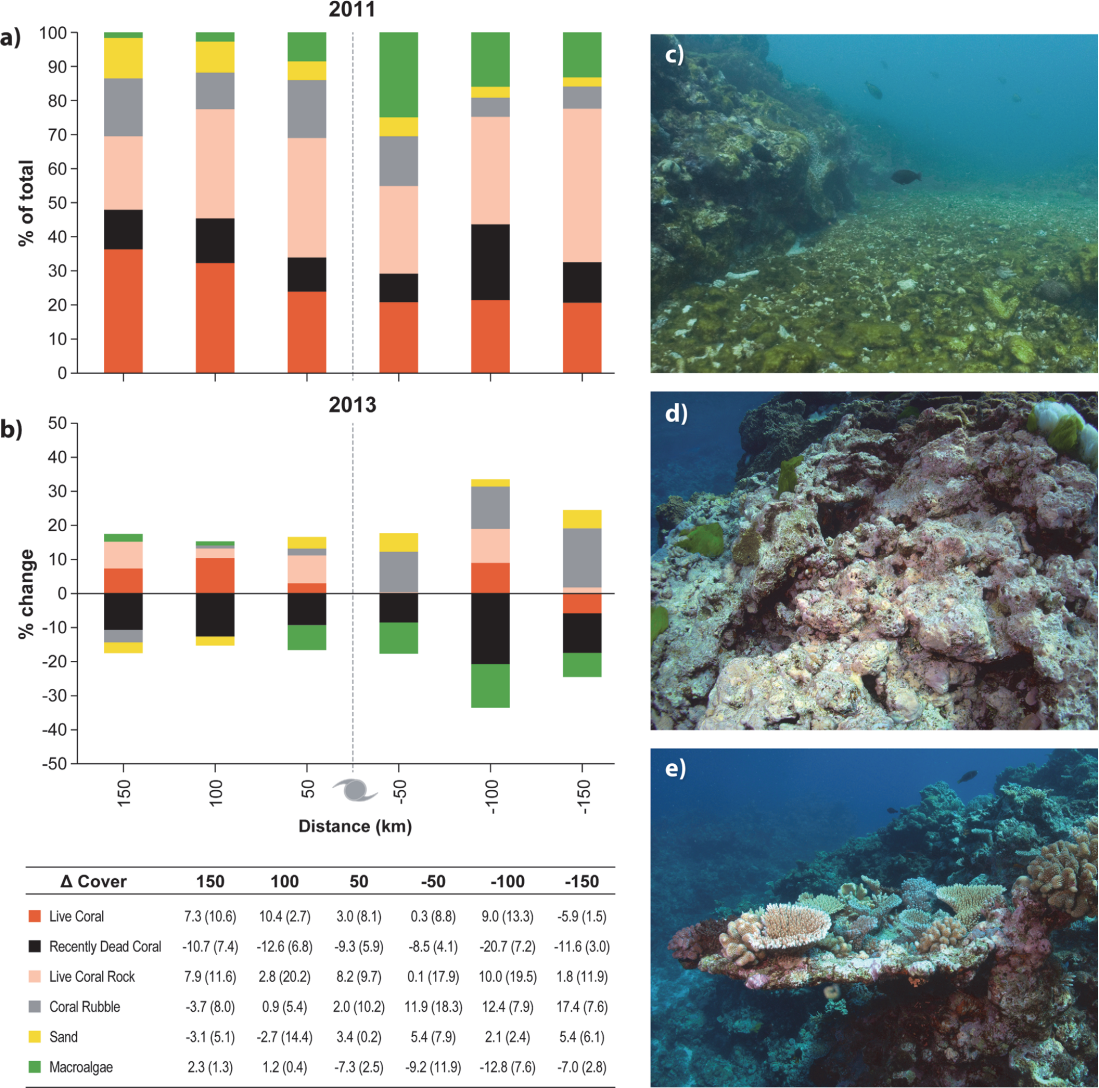
Changes in benthic cover between 2011 and 2013 based on recovery surveys from a sub-set of reefs surveyed 150 km north to 100 km south of the cyclone track (Fig. 2 for reef locations). (a) Proportion of the benthos made up by live coral, recently dead coral, live coral rock, coral rubble, sand and macroalgae. (b) Positive (increasing) and negative (decreasing) percent changes between 2011 and 2013 are shown for each benthic cover category. Average values for change presented in (b) are shown in the table for each segment and benthic cover category with standard deviation values in brackets. Photographs on the right show (c) algae blooms following TC Yasi in 2011, (d) the transition from recently dead coral to live coral rock, and (e) coral recruitment and recovery at Helix Reef.

During the impact assessment surveys extensive algal growth was observed on many of the damaged reefs. Green filamentous algae were observed growing over remnant coral fragments and injured colonies, and blanketing large areas of damaged reef substrate. Dense algal growth was seen on reefs up to 200 km south of the cyclone track (Fig. 9C). The morphology of the algal growth varied with depth, taking the form of a low mat on the mid and lower reef slope, and dense stands of long filaments on the upper slope and reef flat. During the impact assessment algae cover varied from 6.65% (100 km north) to 34.50% (200 km south of the track).

We observed early signs of recovery in the years between 2011 and 2013. Coral cover increased on average in all segments from 150 km north to 100 km south and was >9% in the segments 100 km north and 100 km south of the cyclone eye. Coral cover declined (-5.9±1.5% (sd)) in the segment 150 km south of the track. Between 2011 and 2013, the average change (across all reefs re-surveyed) in coral cover from 150 km north to 150 km south of the track was 4.4% (±9.25%, sd) or ~2% per year. Algae cover declined greater than 7% in all 4 segments between 50 km north and 150 km south and changed <3% 100 and 150 km north of the track. Recently dead coral in 2011, which included recently created ‘rubble’, declined between 8.5% (50 km south of track) and 20.5% (100 km south of track). Some of this space transitioned to live coral, though most transitioned to live coral rock, or stayed as coral rubble (but lost the ‘recently dead’ classification, Fig. 9B).

The rate of coral cover increase we observe per year is less than the rate reported in Emslie et al. [35] between 1993 and 2000 for the Cooktown/Lizard and Capricorn Bunker sectors of the GBR. During those years, coral cover increased 6–7% per year from ~15% in 1993 to ~60% in 2000, at which point coral cover mostly stabilised for the 5 years that followed [35]. Those researchers saw commensurate declines in algae cover as coral cover increased, which we also observed between 2011 and 2013. As an annual rate, the 6–7% per year that Emslie et al. [35] document is ~3 times greater than the ~2% per year rate of recovery we observed between 2011 and 2013. However, in a recent study, the AIMS long-term monitoring program team reported a per year coral cover increase rate of 3.9% for disturbance-free years [36], slightly less than twice the rate we observe. Modelled recovery rates for 1989 to 1994 of 2.5–3.5% [37] are closer to the 2% per year increases we observe. There are probably two causes for the difference between the coral cover rate we document and rates published for the 2 decades preceding TC Yasi. Firstly, structural damage was extensive during TC Yasi and locations with structural damage may take decades to centuries to recover [2,5,6]. This is in keeping with the results from similar surveys presented by Halford et al. [38] who observe exponential differences in recovery rates when comparing the first 5 years after a cyclone with the 5–10 year period. Secondly, an outbreak of crown-of-thorns starfish (COTS, *Acanthaster planci*) started during the recovery period within the area of the GBR where our recovery surveys took place, which is where damage from TC Yasi was most severe. This COTS outbreak is still ongoing at the time of writing. De’ath et al. [17] estimate rates of increase in coral cover in the absence of cyclones, COTS and bleaching to be 2.85% per year during recent decades, which is only 0.7% greater than what we observe. In this sense, we observed strong recovery given both years in our recovery study period were not disturbance free. It is encouraging that we observed increases in coral cover in the years since TC Yasi’s crossing of the GBRMP but the low coral cover increase rate we see of 2% per year is also indicative of the challenges for reefs in recovering from cumulative disturbances.

The recent spate of intense TCs crossing the GBRMP (TCs Ingrid 2005; Monica 2006; Larry 2006; Hamish 2009; Ului 2010; Yasi 2011; Ita 2014) is in stark contrast to the extreme rarity of such TCs from 1970 to 2003. No TCs crossed the GBRMP at greater than category 1 on the Saffir-Simpson scale (category 3 on the Australian scale) from 1970–2003 [39,40]. This has led to speculation that the intensification of TCs (fewer TCs overall, more of them at higher intensity) projected for the southwest Pacific under global warming [41–44] is already occurring in the GBR region. Holland and Bruyere [45] argue that global warming has already substantially increased the proportion of very intense (Saffir Simpson category 4 and 5) TCs globally and regionally since 1975 but that the rate of intensification of TCs is likely to slow. In contrast, large cyclones did not become more prevalent globally or locally from 1978–2011 [22]. There is currently no evidence to suggest large cyclones will occur more frequently as the climate warms [46]. Further, potential changes to the spatial distribution of TC tracks globally and in the southwest Pacific is uncertain [46]. However, even if large TCs remain rare within the GBR, a greater proportion of TCs are predicted to be intense in the future [46]. This indicates an increased likelihood that the large TCs that do cross the GBR in the coming decades will do so at high intensity—as was the case with TC Yasi.

The impact assessment presented here is the largest-in-scale ever conducted on the GBR following a reef health disturbance. In summary, the results of the impact assessment presented here demonstrate all of the following. (1) Structural damage from TC Yasi extended much further on both sides of the cyclone track than has previously been recorded in field surveys of intense cyclones. Following TC Yasi, structural damage was observed: a) nearly 8 times further from the left and 4 times further from the right sides of the track than was the case in 2005 for TC Ingrid [4], and b) nearly 5 times further from the left and 8 times further from the right sides of the track than was the case in 1990 for TC Ivor [3]. (2) Damage at all severity levels was extremely widespread, but structural damage was patchy within all wind zones, which can facilitate future recovery. (3) Many early signs of recovery have already been observed; algae blooms have subsided, crustose coralline algae are covering dead coral and corals are actively recruiting and recovering, albeit at lower rates than seen in previous decades. Understanding patterns in impact severity and recovery enables managers to target local-scale actions to support reef resilience and recovery and conserve the ecological, social, cultural and economic values provided by coral reefs. Examples of such actions include: crown-of-thorns starfish eradication, active reef restoration and the establishment of special management areas or temporary fishing closures (some of which are ongoing at time of publication in response to TC Yasi’s impacts). The process we present here of assessing impacts and recovery and targeting actions is critically important to the adaptive management cycle [47] (Uychiaoco et al. 2005) and will be of increasing importance as disturbance frequencies increase under climate change.

## References

[1] Scoffin TP. The geological effects of hurricanes on coral reefs and the interpretation of storm deposits. Coral Reefs. 1993;12:203–221.

[2] Harmelin-Vivien ML. The effects of storms and cyclones on coral reefs: a review. Journal of Coastal Research, Special Issue. 1994;12, 211–231.

[3] Done TJ. Effects of tropical cyclone waves on ecological and geomorphological structures on the Great Barrier Reef. Cont Shelf Res. 1992;12, 859.

[4] Fabricius KE, De’ath G, Puotinen ML, Done T, Cooper TF, Burgess SC. Disturbance gradients on inshore and offshore coral reefs caused by a severe tropical cyclone. Limnol Oceanogr. 2008;53, 690–704.

[5] Connell JH, Hughes TP, Wallace CC. A 30 year study of coral abundance, recruitment and disturbance at several scales in space and time. Ecol Monogr. 1997;67 (4): 461–488.

[6] Hughes TP, Connell JH. Multiple stressors on coral reefs: A long-term perspective. Limnol Oceanogr. 1999;44, 932–940.

[7] Hughes TP, Tanner JE. Recruitment failure, life histories, and long-term decline of Caribbean corals. Ecol. 2000;81: 2250–2263.

[8] Coles S, Brown E. Twenty-five years of change in coral coverage on a hurricane impacted reef in Hawai‘i: the importance of recruitment. Coral Reefs. 2007;26:705–717.

[9] Hughes TP. Catastrophes, phase shifts and the large scale degredataion of a Caribbean coral reef. Science. 1994;1547. 10.1126/science.265.5178.1547 17801530

[10] Hughes TP, Rodrigues MJ, Bellwood DR, Ceccarelli D, Hoegh-Guldberg O, McCook L, et al Phase shifts, herbivory and the resilience of coral reefs to climate change. Curr Biol. 2007;17(4): 360–365. 17291763 10.1016/j.cub.2006.12.049

[11] Hughes TP, Graham NAJ, Jackson JBC, Mumby PJ, Steneck RS. Rising to the challenge of sustaining coral reef resilience. Trends Ecol Evol. 2010;25, 633–642. 10.1016/j.tree.2010.07.011 20800316

[12] Graham NAJ, Nash KM, Kool TJ. Coral reef recovery dynamics in a changing world. Coral Reefs. 2011;30:283–294.

[13] McClanahan TR, Polunin NVC, Done TJ. Resilience of Coral Reefs In: Gunderson LH and Pritchard L (eds.) Resilience and the Behavior of Large-Scale Systems. 60 ed. London: Island Press 2002.

[14] Carrigan AD, Puotinen ML. Assessing the potential for tropical cyclone induced sea surface cooling to reduce thermal stress on the world’s coral reefs, Geophys Res Lett. 2011;38, L23604, 10.1029/2011GL049722

[15] Woodley JD, Chornesky EA, Clifford PA, Jackson JBC, Kaufman LS, Knowlton N, et al Hurricane Allen's Impact on Jamaican Coral Reefs. Science. 1981;214 (4522): 749–755. 17744383 10.1126/science.214.4522.749

[16] Nott J, Hayne M. High frequency of ‘super-cyclones’ along the Great Barrier Reef over the past 5,000 years. Nature. 2001;413:508–512. 11586356 10.1038/35097055

[17] De'ath G, Fabricius KE, Sweatman H, Puotinen ML. The 27-year decline of coral cover on the Great Barrier Reef and its causes. Proc Nat Acad Sci USA. 2012;109, 17995–17999. 10.1073/pnas.1208909109 23027961 PMC3497744

[18] Holland GJ. An analytic model of the wind and pressure profiles in hurricanes. Monthly Weather Review. 1980;108, 1212–1218.

[19] Young IR. A review of the sea state generated by hurricanes. Marine Structures. 2003;16: 201–218.

[20] Powell MD, Reinhold TA. Tropical cyclone destructive potential by integrated kinetic energy. Bulletin of the American Meteorological Society. 2007;88, 513–526.

[21] Merrill RT. A comparison of large and small tropical cyclones. Monthly Weather Review. 1984;112, 1408–1418.

[22] Knaff JA, Longmore SP, Molenar DA. An Objective Satellite-Based Tropical Cyclone Size Climatology. J Clim. 2014;27, 455–476.

[23] Gardner TA, Cote IM, Gill JA, Watkinson AR. Hurricanes and Caribbean coral reefs: impacts, recovery patterns, and role in long-term decline. Ecol. 2005;86, 174–184.

[24] Manzello DP, Brandt M, Smith TB, Lirman D, Hendee JC, Nemeth RS. Hurricanes benefit bleached corals. Proc Acad Nat Sci USA. 2007;104, 12035–12039. 17606914 10.1073/pnas.0701194104 PMC1924587

[25] Edwards HJ, Elliott IA, Eakin CM, Irikawa A, Madin JS, McField M, et al How much time can herbivore protection buy for coral reefs under realistic regimes of hurricanes and coral bleaching? Glob Change Biol. 2011;17, 2033–2048.

[26] Australian Bureau of Meteorology. Severe Tropical Cyclone Yasi, http://www.bom.gov.au/cyclone/history/yasi.shtml, 2011. Accessed 5 September 2014.

[27] Beeden RJ, Turner MA, Dryden J, Merida F, Goudkamp K, Malone C, et al. Rapid survey protocol that provides dynamic information on reef condition to managers of the Great Barrier Reef, Environ Monit Assess, 2014;1–14, 10.1007/s10661-014-4022-0 25179944

[28] Lukoschek V, Cross P, Torda G, Zimmerman R, Willis BL. The Importance of Coral Larval Recruitment for the Recovery of Reefs Impacted by Cyclone Yasi in the Central Great Barrier Reef. PLoS ONE. 2013;8(6): e65363 10.1371/journal.pone.0065363 23755223 PMC3673992

[29] Perry CT, Smithers SG, Kench PS, Pears B. Impacts of Severe Tropical Cyclone Yasi on nearshore, terrigenous sediment-dominated reefs of the central Great Barrier Reef, Australia. Geomorphology. 2014;222: 92–105

[30] Hayne M, Chappell J. Cyclone frequency during the last 5000 years at Curacoa Island, North Queensland, Australia. Palaeogeography, Palaeoclimatology, Palaeoecology. 2001;168:207–219.

[31] Young IR, Hardy TA. Measurement and modelling of tropical cyclone waves in the Great Barrier Reef. Coral Reefs. 1993;12, 85–95.

[32] Monismith SG. Hydrodynamics of Coral Reefs. Annu Rev Fluid Mech. 2007;39, 37–55.

[33] Madin JS, Connolly SR. Ecological consequences of major hydrodynamic disturbances on coral reefs. Nature. 2006;444, 477–480. 17122855 10.1038/nature05328

[34] Madin JS, Baird AH, Dornelas M, Connolly SR. Mechanical vulnerability explains size-dependent mortality of reef corals. Ecol Lett. 2014; 10.1111/ele.12306 PMC4145665 24894390

[35] Emslie MJ, Cheal AJ, Sweatman H, Delean JSC. Recovery from disturbance of coral and reef fish communities on the Great Barrier Reef, Australia. Marine Ecology-Progress Series. 2008;371:177–190.

[36] Osborne K, Dolman AM, Burgess SC, Johns KA. Disturbance and the dynamics of coral cover on the Great Barrier Reef (1995–2009). PLoS One. 2011;6(3), e17516 10.1371/journal.pone.0017516 21423742 PMC3053361

[37] Done TJ, DeVantier LM, Turak E, Fisk DA, Wakeford M, van Woesik R. Coral growth on three reefs; development of recovery benchmarks using a space for time approach. Coral Reefs. 2010;29:815–833.

[38] Halford A, Cheal AJ, Ryan D, Williams DM. Resilience to large-scale disturbance in coral and fish assemblages on the Great Barrier Reef. Ecol. 2004;85:1892–1905.

[39] Puotinen ML, Done TJ, Skelly WC. An atlas of tropical cyclones in the Great Barrier Reef Region, 1969–1997. CRC Reef Research Centre, Technical Report No. 19. Townsville; CRC Reef Research Centre, 203 pp. 1997.

[40] Puotinen ML. Modelling the risk of cyclone wave damage to coral reefs using GIS: a case study of the Great Barrier Reef, 1969–2003. International Journal of Geographical Information Science. 2007;21, 97–120.

[41] Abbs D. The impact of climate change on the climatology of tropical cyclones in the Australian region. CSIRO Climate Adaptation Flagship Working paper No. 11. CSIRO, Canberra. 2011.

[42] Leslie LM, Karoly DJ, Leplastrier M, Buckley BW.Variability of tropical cyclones over the southwest Pacifi using a high-resolution climate model. Meterol Atmos Phys. 2007;97, 171–180.

[43] Walsh KJE, Nguyen KC, McGregor JL. Fine-resolution regional climate model simulations of the impact of climate change on tropical cyclones near Australia. Clim Dyn. 2004;22, 47–56

[44] CSIRO—Australian Bureau of Meteorology. *Climate change in Australia*: *technical report 2007*. CSIRO. 2007;148 pp.

[45] Holland G, Bruyere CL. Recent intense hurricane response to global climate change. Climate Dynamics. 2014;42 (3–4): 617–627. 10.1521/pdps.2014.42.3.423.The 25211432

[46] Knutson TR, McBride JL, Chan J, Emanuel K, Holland G, Landsea C, et al Tropical cyclones and climate change. Nat Geosci. 2010;3, 157–163.

[47] Uuchiaoco AJ, Arceo HO, Green SJ, Margarita T, Gaite P, Alino PM. Monitoring and evaluation of reef protected areas by local fishers in the Philippines: tightening the adaptive management cycle. Biodiversity & Conservation. 2005;14: 2775–2794.

